# Deciphering the immunocellular regulatory network in inflammatory bowel disease: from susceptibility genes to cellular effectors and toward precision therapies

**DOI:** 10.3389/fimmu.2025.1719366

**Published:** 2026-01-27

**Authors:** Zhuzhu Wu, Xiaolin Wang, Zitong Guan, Mengxue Han, Wenke Ma, Jie Li, Shuai Man, Zhenguo Wang, Qibiao Wu

**Affiliations:** 1 Faculty of Chinese Medicine and State Key Laboratory of Mechanism and Quality of Chinese Medicine, Macau University of Science and Technology, Macau, China; 2 Key Laboratory of Traditional Chinese Medicine Classical Theory, Ministry of Education, Shandong University of Traditional Chinese Medicine, Jinan, China; 3 Institute of Chinese Medical Literature and Culture, Shandong University of Traditional Chinese Medicine, Jinan, China; 4 Zhuhai M.U.S.T. Science and Technology Research Institute, Guangdong-Macao In-Depth Cooperation Zone in Hengqin, Zhuhai, China

**Keywords:** Crohn’s disease, inflammatory bowel disease, personalized medicine, predictive markers, susceptibility genes, ulcerative colitis

## Abstract

Inflammatory bowel disease (IBD) is a chronic, immune-mediated intestinal disorder driven by dysregulated immune responses in genetically susceptible individuals. Despite recent advances in treatment, more than 30% of patients either fail to respond initially or lose response over time, underscoring the need for a deeper mechanistic understanding of immunogenetic pathways and the development of individualized therapeutic strategies. We first discuss how newly identified susceptibility genes (e.g., IL23R, NOD2, BDNF, SLC) and their polymorphisms influence immune cell function and epithelial barrier integrity. Single-cell technologies have further revealed novel cell subsets and interactions underlying disease heterogeneity. We then explore the clinical efficacy of classical and emerging targeted therapies, including cytokine-specific biologics, JAK inhibitors, and novel strategies aimed at restoring regulatory T-cell function or blocking integrin-mediated lymphocyte trafficking. Additionally, we highlight promising therapeutic approaches such as fecal microbiota transplantation, microbial metabolite-based interventions, and nanotherapeutics. We further discuss how genetic insights and immune biomarkers can facilitate treatment personalization and improve prognostic stratification. Ultimately, this review emphasizes the transition from broad immunosuppression to precision medicine and proposes integrated approaches—combining multiomics profiling, immune monitoring, and novel therapeutics—to achieve sustained remission and improve long-term outcomes in IBD patients.

## 1 Introduction

Inflammatory bowel disease (IBD), comprising Crohn’s disease (CD) and ulcerative colitis (UC), is a chronic, relapsing gastrointestinal disorder resulting from immune dysregulation in genetically susceptible individuals upon exposure to environmental and microbial triggers (1). Clinical manifestations include abdominal pain, diarrhea, the passage of mucus or blood in the stool, and weight loss (2). Although the incidence in developed countries has plateaued, the prevalence remains high (3). In contrast, newly industrialized nations are experiencing a rising incidence of IBD. This trend imposes a growing burden on healthcare systems and significantly influences patients’ quality of life, highlighting the urgent need for a deeper mechanistic understanding and the development of transformative therapeutic strategies (4).

The pathogenesis of IBD is characterized by the disruption of intestinal immune homeostasis, wherein aberrant immune activation and impaired resolution of inflammatory responses result in persistent tissue damage (5). Dysfunctional immune cells are central to this process, integrating genetic susceptibility, microbial dysbiosis, and environmental factors to sustain chronic inflammation. Genome-wide association studies (GWASs) have been instrumental in delineating the polygenic architecture of IBD, identifying over 240 risk loci that converge on pathways involved in immune regulation, epithelial barrier integrity, and host–microbe interactions (6, 7). Recent advances in fine-mapping, functional genomics, and single-cell RNA sequencing (scRNA-seq) have further refined these associations, revealing novel susceptibility genes that influence immune cell phenotypes and functions. Concurrently, the therapeutic landscape has expanded from first-generation anti-TNF-α agents to include next-generation biologics and oral small molecules that target specific immune pathways with greater precision.

Despite these advancements, several challenges persist. Disease heterogeneity—driven by diverse immune cell states across subtypes, disease phases, and anatomical locations—remains poorly defined. The functional implications of newly identified risk alleles for immune cell differentiation, metabolism pathways, and effector functions are still not fully understood. Furthermore, over 30% of patients experience either primary nonresponse or secondary loss of response to current therapies, reflecting gaps in our understanding of the adaptive immune mechanisms underlying treatment resistance (8).

This review synthesizes recent progress in immune-regulatory networks central to IBD pathogenesis, focusing on three key areas: (1) genetic blueprinting—how newly discovered susceptibility genes (2022–2025) reshape immune cell signaling, epigenetic regulation, and functional responses; (2) cellular effectors—the roles of emerging immune cell subsets and their interactions within the tissue microenvironment, as elucidated by scRNA-seq and spatial omics technologies; and (3) therapeutic translation—the mechanisms, efficacy, and limitations of the latest targeted therapies, linking clinical outcomes to underlying immune cell biology. By integrating insights from immunogenetics, cellular immunology, and clinical pharmacology, this review aims to outline a roadmap toward precision immunomodulation in IBD, where genetic and immune cell-derived biomarkers may serve as key tools for patient stratification and for guiding the development of targeted therapeutic strategies.

## 2 The immunogenetic basis of IBD: susceptibility genes and their role in immune function

### 2.1 Genetic architecture of IBD: from GWAS to fine-mapping

GWAS represents a foundational approach in complex disease genetics, enabling the systematic identification of genetic variants associated with diseases by comparing genomic profiles between large cohorts of affected individuals and healthy controls. GWASs conducted in Asian populations have revealed novel IBD risk loci not observed in European cohorts, such as the UC-associated variant rs76227733 in the LCOR–SLIT1 region at 10q24 and the CD-linked variant rs2240751 in the MFSD12-C19orf71-FZR1-DOHH region at 19p13, highlighting the importance of population-specific genetic backgrounds in shaping disease susceptibility (9). Furthermore, GWASs have implicated numerous noncoding variants implicated in IBD pathogenesis, many of which are likely to influence disease onset and progression through modulation of gene expression. However, GWASs are designed primarily to detect common genetic variants with minor allele frequencies (MAFs) typically exceeding 1%, leaving many rare variants (MAFs <1%) undetected due to insufficient statistical power. Additionally, the association signals identified by GWAS often reflect linkage disequilibrium rather than direct causality, necessitating subsequent fine-mapping to pinpoint the true functional variants.

Fine-mapping is a critical step following GWAS aimed at narrowing associated genomic regions to identify likely causal variants. By integrating high-density genotyping data with advanced statistical models, this approach refines association signals to a small set of candidate variants, thereby enabling more precise functional hypotheses (10). For example, fine-mapping in African ancestry populations at the PTGER4 locus narrowed the candidate set to 22 single nucleotide polymorphisms (SNPs) (11). Experimental validation via massively parallel reporter assays (MPRA) has further confirmed causal variants, such as those at the ITGA4 locus, elucidating their role in IBD pathogenesis (12). Collectively, these approaches illustrate how fine-mapping bridges the gap between genetic and biological mechanisms, facilitating downstream functional studies and potential clinical translation.

The integration of GWAS findings with functional genomic approaches—such as expression quantitative trait locus (eQTL) mapping, MPRA, and cross-disease colocalization analyses—has revealed key immunoregulatory pathways involved in IBD (13). Transcriptome-wide association studies (TWASs) have revealed genetic overlap between IBD and psychiatric disorders, implicating genes involved in the brain–gut axis in a cohort of 180,592 individuals with gut inflammation or psychiatric conditions (14). Moreover, context-specific eQTL analyses have identified 190 inflammation-dependent regulatory variants in mucosal tissues from a cohort of 171 IBD patients (15). Combined linkage and fine-mapping approaches involve the localization of UC-associated regulatory mechanisms to the chromosomal region 7p22.3–7p15.3 in a Danish familial linkage study (16). Additionally, QTL analyses have linked genetic variation to the gut microbiome composition in IBD patients (17, 18), and Mendelian randomization studies have elucidated shared genetic architectures between IBD subtypes (19, 20). Despite these advances, GWAS-identified loci account for only a fraction of IBD heritability, underscoring the contributions of rare variants, epigenetic regulation, and gene–environment interactions (21, 22). A major ongoing challenge remains the functional annotation of noncoding risk variants. Future research should focus on elucidating causal molecular mechanisms, clarifying shared pathophysiologies across diseases, and translating genetic discoveries into clinical applications.

### 2.2 Susceptibility genes involved in core pathways of IBD

**Figure 1:**
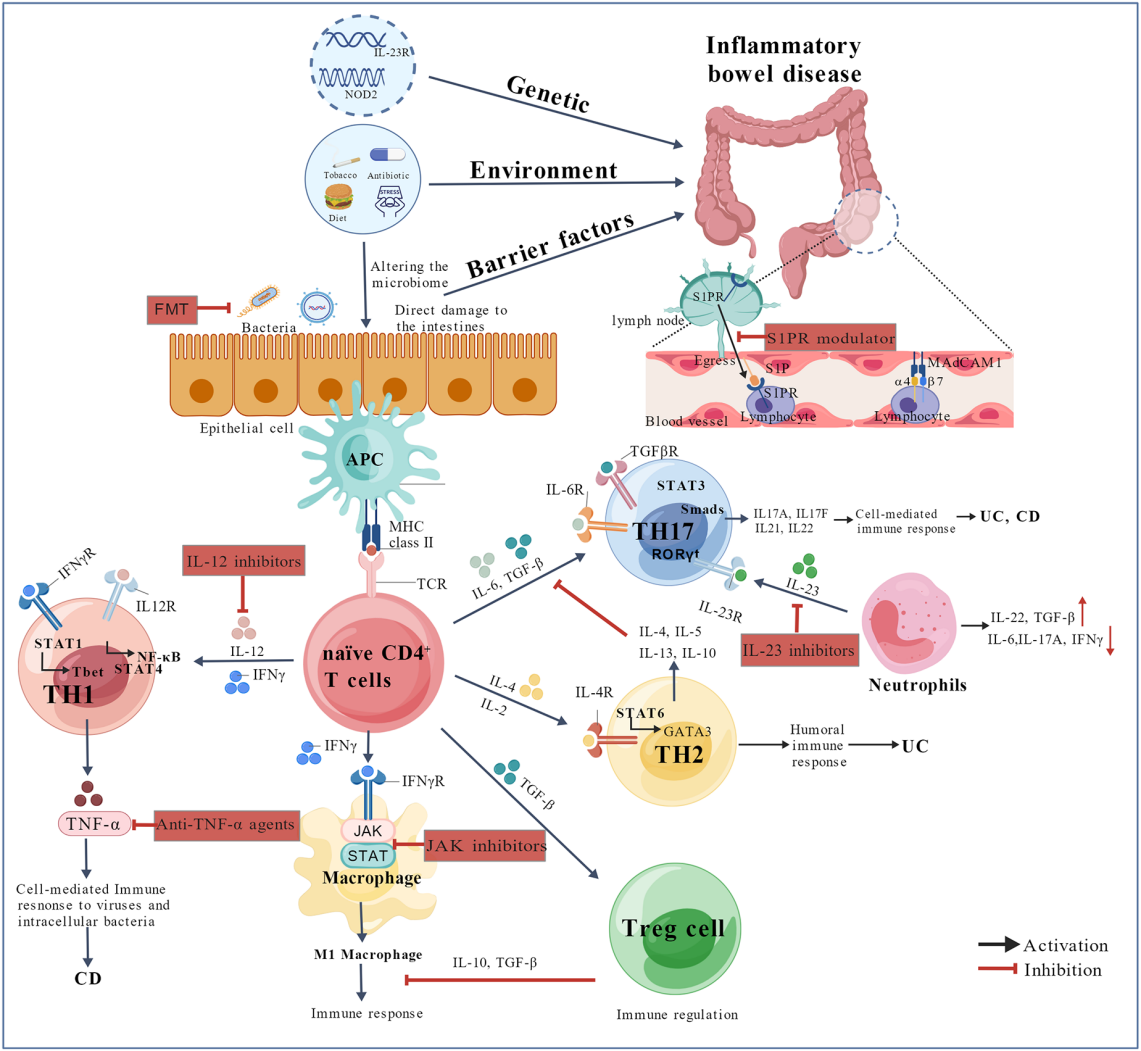
Pathophysiology and emerging therapeutic strategies in IBD. Under the influence of environmental factors, pathogens or alterations in the gut microbiota can disrupt intestinal mucosal barrier function or directly activate immune pathways in genetically susceptible individuals. Within the mucosal immune compartment, APCs present microbial antigens via MHC molecules to the TCR, initiating T-cell activation. In the presence of polarizing cytokines such as IL-23, TGF-β, and IL-6, naïve CD4^+^ T cells differentiate into proinflammatory Th17 cells. A dysregulated cytokine environment further amplifies the inflammatory cascade, driving the activation of Th17 and ILC3 cells. This cross-talk between innate and adaptive immunity, which is mediated by cytokines including TNF-α, IL-6, IL-1β, and IFN-γ, perpetuates chronic intestinal inflammation. Combining these mechanistic insights with cytokine-targeted therapies (e.g., anti-IL-12/23 agents), JAK inhibitors, anti-integrin therapies, and fecal microbiota transplantation offers promising approaches for modulating immune responses and improving clinical outcomes in IBD. APC, antigen-presenting cell; CD, Crohn’s disease; DCs, dendritic cells; FMT, fecal microbiota transplantation; IBD, inflammatory bowel disorder; IFN-γ, interferon-gamma; IL, interleukin; ILCs, innate lymphoid cells; JAK, Janus kinase; MHC, major histocompatibility complex; MAdCAM-1, mucosal addressin cell adhesion molecule-1; NF-κB, nuclear factor kappa-light-chain-enhancer of activated B cells; NOD2, nucleotide-binding oligomerization domain 2; RORγt, retinoic acid receptor-related orphan receptor gamma t; STAT, signal transducer and activator of transcription; TCR, T-cell receptor; TGF-β, transforming growth factor-beta; TNF-α, tumor necrosis factor-alpha; Th1/Th2/Th17, T helper 1/T helper 2/T helper 17 cells; Tregs, regulatory T cells; TRM, tissue-resident memory T cells; UC, ulcerative colitis. This construct was created with BioGDP.com (23).

The immunogenetic profile of IBD encompasses pathways involved in innate and adaptive immunity, epithelial barrier function, and neuroimmune–metabolic axes. Susceptibility genes contribute to pathogenesis and responses to environmental triggers (e.g., a proinflammatory diet, stress, antibiotic use, and smoking) through cell-specific mechanisms (e.g., macrophage polarization and Th17 differentiation). **Figure 1** illustrates pathophysiology and emerging therapeutic strategies in IBD.

#### 2.2.1 Innate immunity and barrier integrity

Gut macrophages and dendritic cells (DCs) recognize microbial signals through pattern recognition receptors (PPRs), thereby modulating inflammatory cascades and epithelial barrier function (24). Nucleotide-binding oligomerization domain 2 (NOD2), a pivotal PPR, detects bacterial muramyl dipeptide, and its dysregulation is mechanistically linked to CD pathogenesis. NOD2 also promotes intracellular pathogen clearance through autophagy activation. In the lipopolysaccharide-treated BV2 microglial cell line, the binding of high mobility group box-1 (HMGB1) protein with NOD2 and autophagy-related 16-like 1 (ATG16L1) was significantly enhanced, and this binding was consistent with the induction time of LC3II (an autophagy marker), suggesting that NOD2 participates in immune regulation through the autophagy pathway mediated by HMGB1 (25). In murine intestinal epithelial cell models, the ELMO1-NOD2 interaction is essential for defense against adherent-invasive E. coli (AIEC) (strain LF82) associated with CD (26). NOD2 mutations (rs2066844, rs2066845, and rs2066847) impair pathogen clearance and exacerbate inflammatory responses in CD (27).

ATG16L1, an essential autophagy effector, carries the T300A coding variant—a major genetic risk factor for CD (27). The ATG16L1 polymorphism (T300A; rs2241880) raises the CD risk by disrupting C-terminal WD40 domain interaction and impairing non-canonical autophagy (28). ATG16L1-deficient mice show worse epithelial injury, stronger inflammation, and lower survival in T-cell-mediated colitis, indicating its role in maintaining epithelial integrity through autophagy (29). In colon cancer organoids, ATG16L1 loss increases cell death induced by tumor necrosis factor-alpha (TNF-α) and interferon-gamma (IFN-γ) and reduces cancer stem cells, suggesting that autophagy affects tumor progression by regulating immune pressure (30).

Epithelial barrier dynamics are regulated by the autophagy-dependent degradation of tight junction proteins, including the pore-forming protein claudin-2 (CLDN2) and occludin. Autophagy enhances the barrier by degrading CLDN2, whereas loss of ATG16L1 leads to CLDN2 accumulation and exacerbates intestinal permeability, facilitating antigen translocation and inflammation (31, 32). Barrier disruption initiates a pathogenic cascade: exposed antigens activate lamina propria immune cells (e.g., macrophages), which release IFN-γ and TNF (30). These cytokines further compromise barrier function and recruit neutrophils, amplifying inflammation. These interactions highlight a complex interplay among autophagy, barrier-associated molecules (e.g., CLDN2, caspase-8, occludin), and immune cells (including BECN1/Beclin-1-influenced macrophage polarization) that collectively shape intestinal inflammation (33, 34). Therefore, therapeutic strategies aimed at modulating autophagy or selectively inhibiting pore-forming claudin-2 may hold promise for the IBD treatment.

Hepatocyte nuclear factor 4α (HNF4α), a conserved nuclear receptor in intestinal epithelial cells (IECs), regulates barrier integrity and immune homeostasis by driving the expression of immunomodulatory molecules (35). This pathway facilitates crosstalk with intraepithelial lymphocytes (IELs), promoting γδ T-cell differentiation and immune tolerance (35). Crucially, HNF4α maintains tight junction architecture and restricts microbial translocation. Its deficiency in IBD compromises epithelial sealing, leading to local immune dysregulation characterized by elevated TNF-α and interleukin (IL)-6, along with macrophage and T-cell activation (36). Barrier disruption allows microbial access to the lamina propria, triggering toll like receptor (TLR)/nuclear factor-κB (NF-κB) signaling and neutrophil infiltration (37). Concurrent cytokine dysregulation stimulates excessive IL-22 production and JAK-STAT hyperactivation, intensifying mucosal inflammation (38). HNF4α deficiency also reprograms immune responses, promoting IL-17-driven pathology while impairing anti-inflammatory macrophage polarization (39, 40). Therapeutically, HNF4α dysfunction correlates with impaired mucosal healing, highlighting its potential as a target. Restoring the HNF4A-BTNL axis may reinforce IEL-mediated tolerance and barrier function in DSS-induced colitis mice (35).

#### 2.2.2 IL-23/Th17 axis

IL-23 is predominantly secreted by DCs and macrophages following microbial stimulation. Upon binding to the IL-23 receptor (IL-23R), it promotes the differentiation of pathogenic Th17 cells, activates STAT3 phosphorylation via the JAK/STAT signaling pathway, and enhances the production of IL-17A and IFN-γ, thereby driving intestinal inflammation and fibrosis (41). IL-23R is highly expressed on Th17 cells, and is linked to IBD susceptibility and severity (42). The IL23R variants (rs11209026, rs76418789, and rs41313262) have been shown to impair IL-23-mediated Th17 differentiation, leading to reduced frequencies of Th17 cells in peripheral blood, decreased IL-17A secretion, and a diminished inflammatory response (43). In contrast, the IL-23R rs10889677 polymorphism is associated with an increased risk of CD and UC (41).

Janus kinase 2 (JAK2) and tyrosine kinase 2 (TYK2) mediate IL-23 signal transduction, critically regulating Th1/Th17 differentiation and the production of proinflammatory cytokines (TNF-α and IFN-γ). This amplifies the inflammatory effect, leading to persistent inflammation in IBD. The JAK2 V617F mutation constitutively activates JAK-STAT signaling, potentially amplifying IL-23-driven inflammation and intestinal injury. JAK inhibitors alleviate the inflammatory response of IBD by inhibiting the JAK-STAT signaling pathway. TYK2 polymorphisms (rs34536443, rs35018800, rs2304256, and rs12720356) decrease TYK2 activity and function; reduce IL-23-dependent JAK/STAT signaling; weaken mucosal immunity; and are associated with protection against IBD (44). In T-cell transfer-induced colitis models, TYK2 inhibitors alleviate disease severity by suppressing Th1 differentiation and reducing IL-23 responsiveness (45).

STAT3 is a key transcription factor required for Th17 cell differentiation. STAT3 serves as a central mediator of IL-23 signaling, and its phosphorylation (p-STAT3) is directly involved in the differentiation and functional maintenance of Th17 cells. According to a recent report, IL-23 stabilizes Th17 cells through the IL-12RB1/TYK2/STAT3 axis, and disruption of this pathway suppresses Th17−driven inflammatory responses in mice with colitis (46). Furthermore, STAT3 also mediates the restorative effect of Th17−derived IL−22 on the intestinal epithelium, whereas its excessive activation contributes to sustained mucosal inflammation (47, 48). A recent study revealed that STAT3 imposes self-directed regulation during the specific differentiation of Th17 cells by controlling the transcription of Hipk2 (49). STAT3 deficiency or dysfunction impairs Th17 responses and ameliorates T-cell-mediated colitis (47), whereas gain-of-function mutations increase Th17 plasticity and proinflammatory conversion (50). Despite STAT3 enrichment in active IBD mucosa, impaired IL-23-induced pSTAT3 nuclear translocation results in reduced IL-17A and IL-22 secretion (47). STAT3-associated transcriptional signatures may serve as biomarkers to stratify treatment responses, enabling precision targeting of this pathway. Retinoic acid receptor-related orphan receptor gamma t (RORγt), encoded by RORC, sustains pTh17 plasticity through T-bet coexpression, thereby facilitating IL-23-driven inflammatory phenotypes (51). Its expression is correlated with elevated IL-23R levels, amplifying pathogenic signals. RORγt also regulates T cell stability and governs ILC3 differentiation (52). IL-10 derived from ILCs suppresses macrophage activation, highlighting the complex interplay between innate and adaptive immune responses (53, 54).

#### 2.2.3 TL1A/TNFSF15-DR3 axis

TNF-like ligand 1A (TL1A, TNFSF15), a member of the TNF superfamily, binds to death receptor 3 (DR3) to promote T-cell activation and differentiation, thereby amplifying intestinal inflammation (55). The TNFSF15 risk variant (rs6478109) is associated with increased CD susceptibility, CD-associated fibrosis and structuring (56, 57). This variant increases the expression of profibrotic proteins involved in structuring the CD mucosa. In the T-cell transfer colitis model, TL1A-deficient CD4^+^ T cells fail to polarize into Th1/Th17 cells and do not induce colonic inflammation (58). TL1A localizes to both the cytoplasm and nucleus of DCs, where it enhances antigen uptake and promotes TLR4-mediated DC activation by positively regulating the DC-specific ICAM-grabbing nonintegrin/RAF1/NF-κB signaling pathway, thereby driving the differentiation of naïve CD4^+^ T cells into Th1 and Th17 cells in colitis mouse models (58). Clinically, an elevated platelet-to-lymphocyte ratio (PLR) and neutrophil-to-lymphocyte ratio (NLR) during active IBD reflect TL1A-driven lymphocyte redistribution (59).

#### 2.2.4 IFN-γ signaling axis

IFN-γ, a signature Th1 cytokine that is overexpressed in CD, drives epithelial inflammation (60). TYK2-dependent IFN-response genes are correlated with IBD activity (61). Impaired IFNγRα signaling in IECs, caused by N-glycosylation-dependent instability, compromises tumor surveillance and predicts poor colorectal cancer prognosis (62). Paradoxically, IFN-γ upregulates TNFR2 to promote mucosal repair (63), whereas CBX3 (HP1γ) epigenetically suppresses IFN-γ-responsive genes (STAT1 and CD274), thereby disrupting gut homeostasis (64). A pathogenic CCR5^+^ Th17 subset co-expressing T-bet and RORγt emerges in CD, coproducing IL-17A and IFN-γ to drive inflammation (51). Analysis of gastrointestinal mucosal biopsies from pediatric patients shows that IFNγ expression is elevated in both ileum and colon in CD but only in the colon in UC, offering a potential immunopathological basis for distinguishing CD from UC (65). IFN-λ (type III IFN) exerts dual effects—promoting colonic epithelial repair while exacerbating ileal apoptosis due to restricted receptor expression (66). Collectively, these findings highlight the central role of IFN-γ signaling in linking epithelial integrity, pathogenic lymphocyte subsets, and genetic susceptibility in IBD, suggesting potential diagnostic biomarkers (IFN-γ, CCR5^+^ Th17) and therapeutic targets (IFN-λ receptor modulation).

#### 2.2.5 Neuroimmune-metabolic axis

Brain-derived neurotrophic factor (BDNF) and solute carrier (SLC) transporters have emerged as key susceptibility genes that regulate neuroimmune–metabolic crosstalk in IBD (67). BDNF is widely expressed in the gastrointestinal tract and regulates intestinal motility, secretion, sensation, immunity, and mucosal integrity. Its expression is regulated by inflammatory factors, neurotransmitters, and the microbiome through multiple signaling pathways. In colitis rats, TNF-α and IL-1β upregulate BDNF protein expression and secretion in colonic smooth muscle via Ca²^+^ and PKA signaling (68). BDNF can promote its own synthesis through ERK and PI3K pathway-mediated positive feedback (69). Gut microbiota also regulate BDNF via the gut–brain axis; for example, butyrate crosses the blood–brain barrier and modulates BDNF expression to enhance synaptic growth and neuroprotection (70).

BDNF has a dual, context-dependent role in IBD. In the inflammatory phenotype, it acts as a neuroimmune modulator by enhancing IgA production, influencing T-cell differentiation, and promoting mucosal repair; early BDNF deficiency impairs healing, suggesting that targeting BDNF signaling may aid mucosal restoration (71). In contrast, in the structuring phenotype, chronically elevated BDNF functions as a pro-fibrotic mediator, driving abnormal neural remodeling, increased nerve fiber density, and collagen deposition leading to intestinal wall thickening and stricture formation (71). The Val66Met polymorphism (rs6265) affects BDNF trafficking and activity-dependent secretion and is linked to psychiatric and metabolic disorders; however, its association with IBD susceptibility or phenotype remains unclear (72). Exogenous BDNF administration inhibits TLR4 signaling and prevents experimental necrotizing enterocolitis in mice, demonstrating its immunomodulatory potential (73). In colitis models, BDNF enhances smooth muscle motility and peristalsis via the TrkB/PLC/IP3 pathway (74). By suppressing IL-4, IL-8, and Fas/FasL-mediated cell death while promoting IL-10, BDNF alleviates colitis symptoms.

IBD patients frequently suffer from sleep disturbances and mood disorders, with BDNF and its precursor proBDNF being critical mediators of brain–gut communication. Sochal M et al. reported reduced serum BDNF mRNA but elevated BDNF protein levels in IBD patients, which correlated positively with sleep efficiency (75). Anti-TNF-α therapy upregulates BDNF mRNA, suggesting that inflammation may worsen neuropsychiatric symptoms via BDNF modulation. Tang et al. demonstrated in a DSS-induced chronic colitis model that cardiac impairment occurs through the IL-1β/miR-155/BDNF axis (76). Inhibiting BDNF signaling could thus protect against heart failure in IBD patients. BDNF may also contribute to IBD comorbidities, such as increased Parkinson’s disease risk. These findings underscore the multifaceted roles of BDNF in IBD: promoting enteric neural repair, enhancing barrier function, and modulating local immune responses. The neural–BDNF–immune axis represents a promising therapeutic target. However, further studies are needed to clarify its mechanisms and therapeutic potential.

Polymorphisms in SLC family genes influence immune cell function and metabolic homeostasis and are closely associated with IBD susceptibility. For example, SLC2A14 encodes GLUT14, a transporter responsible for glucose and dehydroascorbate uptake. Its expression in Paneth cells may regulate intestinal barrier function and immune activity through glucose metabolism. Specific SLC2A14 polymorphisms (e.g., rs2889504-T and rs10846086-G) are associated with increased IBD risk (77). Similarly, SLC39A10 variants (rs529078926 in UC and rs188606584 in CD) also correlate with increased susceptibility (78). T cell-specific SLC39A10 knockout mice exhibit attenuated IBD progression and increased apoptosis via p53/p21- and Bcl2-independent pathways, indicating a role for SLC39A10 in T-cell survival and autoimmunity (78). The SLC26A3-encoded chloride transporter DRA is downregulated in colitis, and its deficiency compromises intestinal barrier function and increases IBD susceptibility (79). Loss of DRA function promotes proinflammatory signaling in immune cells, highlighting its potential as a therapeutic target in UC (79, 80). Additionally, SLC6A14 is significantly upregulated in UC patients and correlated with prostaglandin synthesis-related genes. It may contribute to UC pathogenesis through ferroptosis—an iron-dependent form of regulated cell death—offering novel insights into inflammatory regulation (81). SLC39A8 is a metal ion transporter involved in the regulation of blood manganese (Mn) levels and the pathogenesis of IBD. Briggs K et al. demonstrated that mice with IEC-specific knockout of SLC39A8 (SLC39A8-IEC KO) exhibit markedly reduced Mn levels and impaired epithelial integrity, indicating that SLC39A8 maintains intestinal barrier function via Mn homeostasis (82). Inhibition of alkaline ceramidase 1 (ACER1) improved barrier dysfunction in SLC39A8-IEC KO mice, suggesting that loss of SLC39A8 function may exacerbate IBD progression through disruption of sphingolipid metabolism. Therefore, ACER1 represents a potential therapeutic target in SLC39A8-related IBD. The SLC39A8/ZIP8 variant rs13107325 (A391T) has been shown to reduce Veillonella abundance and plasma FGF19 levels while increasing total bile acid in CD patients, all of which are associated with increased CD risk (83). Future research should further investigate the mechanisms by which SLC genes modulate immune cell functions and explore their potential as therapeutic targets in IBD.

### 2.3 Gene–cell interactions

The pathogenesis of IBD is fundamentally driven by dysregulation of the immune regulatory network. Genetic susceptibility plays a pivotal role in the development, function, and signaling pathways of immune cells. Autophagy-related genes (e.g., NOD2, ATG16L1) impair Paneth cell function and intracellular pathogen clearance, leading to defective microbial sensing and exacerbated Th1/Th17-driven inflammation. Concurrently, hyperactivation of the IL-23/JAK-STAT axis—involving genes such as IL23R, JAK2, TYK2, and STAT3—promotes the differentiation and persistence of pro-inflammatory Th17 cells, while loss-of-function in regulators like PTPN2 reduces control over inflammatory signals. Epithelial barrier integrity is weakened by disrupted tight junctions (e.g., claudin-2), altered metal ion transport (SLC39A8/A10), and impaired epithelial regulation (HNF4α), increasing intestinal permeability. Genetic risk for pro-fibrotic factors like TL1A activates the TGF-β/Smad3 pathway, contributing to the structuring phenotype. Furthermore, immune checkpoint molecules (e.g., PD-L1) and neurotrophic factors (e.g., BDNF) modulate inflammatory crosstalk within the neuro-immune network. These pathways interact and influence each other, collectively shaping individual susceptibility, clinical presentation, and disease progression. **Table 1** lists key genetic determinants in IBD, including susceptibility genes, specific variants, functional roles, underlying immune mechanisms, and associated diseases.

**Table 1:** Genetic determinants in IBD: susceptibility genes, variants, functions, immune mechanisms, and associated diseases.

Gene	Variant(s)	Function	Associated disease	Primary immune cell affected	Key immune mechanism	References
NOD2	rs2066844 (A702T), rs2066845 (G908A), rs2066847 (L1007insC)	Autophagy, microbial sensing	CD	Paneth cells, Th1/Th17 cells	Reduced antimicrobial peptide secretion; excessive Th1/Th17 activation	(27)
ATG16L1	rs2241880 (T300A)	Autophagy	CD	Paneth cells, Th1/Th17 cells	Impaired autophagy; dysregulated cytokine production	(27)
IL23R	rs11209026 (R381Q), rs76418789 (G149R), rs41313262 (V362I)	Pro-inflammatory response	CD, UC	Th17 cells	Reduced IL-23R expression → decreased STAT3 activation → lower IL-17A production	(43, 84)
	rs10889677	Pro-inflammatory response	CD, UC	Th17 cells	Enhanced IL-23R expression → increased STAT3 binding → elevated IL-17A	
JAK2	V617F	Cytokine and growth factor signal transduction	CD, UC	Th1/Th17 cells	Constitutive JAK-STAT activation → enhanced mucosal inflammation	(85)
TYK2	rs34536443 (P1104A), rs35018800 (A928V), rs2304256 (V362P), rs12720356 (I684S)	Cytokine receptor signaling	CD, UC	Th1/Th17 cells	Reduced TYK2 activity → suppressed JAK/STAT signaling → attenuated inflammation	(44)
PTPN2	rs1893217, rs2542151, rs7234029	Tyrosine phosphatase signaling	CD, UC	Macrophages, IECs	Increased claudin-2 and CEACAM6 → enhanced adherent-invasive E. coli colonization → impaired barrier function	(86)
TL1A/TNFSF15	rs6478109	Pro-inflammatory and pro-fibrotic cytokine	CD (structuring)	Macrophages, CD4+/CD8+ T cells	Elevated TL1A → promotes pro-fibrotic protein expression and TGFβ1/Smad3 activation → fibrosis	(58)
IL-10	rs3024505	Anti-inflammatory cytokine signaling	CD, UC	Regulatory cells	Increased IBD risk; impaired anti-inflammatory signaling	(56, 57)
SLC2A14	rs2889504-T, rs10846086-G	Glucose transport	CD, UC	IECs	Altered glucose transport → increased IBD risk	(87)
SLC39A10	rs529078926	Metal ion homeostasis	UC	IECs	Dysregulated metal ion homeostasis → increased UC risk	(78)
	rs188606584	Metal ion homeostasis	CD	IECs	Dysregulated metal ion homeostasis → increased CD risk	
SLC39A8/ZIP8	rs13107325 (A391T)	Manganese transport, bile acid metabolism	CD	Macrophages	Reduced Veillonella abundance → altered bile acid metabolism → decreased FGF19 signaling	(83)
ETS2	rs2836882	Transcription factor, macrophage regulation	CD, UC	Monocytes/Macrophages	ETS2 superenhancer activation → upregulation of MECOM → increased IBD risk	(88, 89)
IL-23	–	Pro-inflammatory response	CD, UC	Myeloid cells, T cells	Promotes Th17 differentiation and stabilization	(43, 84)
HMGB1	–	Autophagy, DNA binding, pro-inflammatory damage-associated molecular pattern	CD, UC	Macrophages, Dendritic cells	Promotes inflammation and autophagy dysregulation	(90)
ELMO1	–	Autophagy, phagocytosis	CD	Phagocytic cells	Regulates bacterial clearance and inflammatory responses	(26)
ULK1	–	Autophagy initiation	CD	Paneth cells, IECs	Core kinase in autophagy initiation; deficiency disrupts intestinal homeostasis	(91)
HNF4α	–	Regulation of epithelial cell junctions	UC	IECs	Regulates epithelial integrity and barrier function	(35)
STAT3	–	Transcription factor for cytokines and growth factors	CD, UC	Multiple immune and epithelial cells	Mediates signals from IL-6, IL-10, IL-22; regulates Th17 differentiation and epithelial repair	(47)
RORγt	–	Th17 cell differentiation	CD, UC	Th17 cells	Master transcription factor for Th17 cell lineage commitment	(51)
STAT1	–	Signal transduction, response to IFNs	CD, UC	Multiple immune cells	Mediates type I and II interferon signaling; modulates inflammatory responses	(64)
CD274 (PD-L1)	–	Immune checkpoint regulation	CD, UC	Antigen-presenting cells, T cells	Inhibits T-cell activation; modulates mucosal immunity	(64)
BDNF	–	Neurotrophic factor, immune modulation	CD, UC	Neurons, immune cells	Modulates neuro-immune interactions and intestinal inflammation	(71)

IBD, inflammatory bowel disease; UC, ulcerative colitis; CD, Crohn’s disease; APCs, antigen-presenting cells; IECs, intestinal epithelial cells.

### 2.4 Gene–environment interplay in IBD pathogenesis

Gene–environment (notably microbiota and diet) interactions shape the host–microbiota interface of IBD. Variations in IBD risk genes (NOD2 and ATG16L1) compromise mucosal barrier integrity and reduce antimicrobial peptide secretion by Paneth cells, ultimately leading to microbial dysbiosis (27). Proinflammatory diets, particularly high-fat diets, disrupt bile acid metabolism and promote the proliferation of pathogenic bacteria and colitis in mice (92). In the gut, the microbiota converts primary bile acids into secondary derivatives, among which deoxycholic acid can induce Paneth cell dysfunction, whereas lithocholic acid derivatives (such as 3-OxoLCA and isoalloLCA) inhibit Th17 cell differentiation and promote Treg cell generation, thereby modulating intestinal immune responses (93). Dietary patterns significantly influence the progression of intestinal inflammation by modulating specific bacterial communities and their metabolic activities. Dietary fiber promotes intestinal motility and microbial balance. Tea extracts can modulate the composition of commensal and pathogenic bacteria, alter microbial metabolism, and potentially alleviate colitis symptoms (94). Additionally, low-fermentation diets and probiotic supplementation may help reduce excessive inflammatory responses in individuals with genetic susceptibility (95–97).

## 3 Functions and dysregulation of key immune regulators: recent advances

### 3.1 Innate immune sentinels

#### 3.1.1 Macrophages

Macrophages mediate intestinal homeostasis through autophagic pathogen clearance and inflammation regulation (98). Macrophages can be classified into resident macrophages (M2) and inflammatory macrophages (M1). M2-type macrophages promote epithelial repair, angiogenesis, and fibrosis. In IBD, microbial triggers drive macrophage polarization toward proinflammatory M1 phenotypes, thereby disrupting tissue homeostasis (98). Mutations in susceptibility genes (ATG16L1, NOD2) in CD patients impair autophagic function, compromising macrophage-mediated pathogen clearance and resolution of inflammation, which exacerbates disease severity (99). Therapeutic interventions targeting macrophage dysfunction show promise; for example, the gp130 inhibitor bazalidixen restructures the myeloid stromal niche and attenuates NOD2-mediated fibrosis in CD (100). Macrophages also engage multiple regulatory pathways, including metabolism, epigenetics, and neural regulation. Lactate metabolism in macrophages influences Treg differentiation, while abnormal lipid metabolism, such as linoleic acid accumulation, promotes inflammation (101). PTPN2 (protein tyrosine phosphatase nonreceptor type 2) deficiency induces autophagy defects, reducing the clearance of AIEC and promoting the inflammation response (102). The risk allele PTPN2 rs1893217 increases susceptibility to AIEC invasion, whereas tofacitinib significantly reduces AIEC colonization in IBD patients (86). Additionally, LIM domain only 7 (LMO7) deficiency exacerbates inflammatory injury through metabolic–epigenetic reprogramming, suggesting that LMO7 or macrophage metabolism may serve as potential therapeutic targets (103). scRNA-seq has revealed novel aspects of macrophage biology, such as opioid signaling-associated monocytes potentially influencing IBD progression via neuroimmune crosstalk (104). scRNA-seq has also revealed two novel macrophage subsets in Chinese patients with UC (105). Furthermore, macrophage RNF128 regulates the inflammatory response through S100A8 ubiquitination, and the cargo receptor Tollip recognizes and mediates S100A8 degradation (106). Targeting the RNF128-Tollip-S100A8 axis may constitute a novel therapeutic strategy for alleviating colitis.

#### 3.1.2 Dendritic cells

As key antigen-presenting cells, DCs initiate innate immunity under homeostasis by inducing T-cell differentiation and maintaining immune tolerance (107). DCs exhibit substantial heterogeneity and include conventional DCs (cDCs), monocyte-derived DCs, plasmacytoid DCs (pDCs), and the recently identified DC3 subset. Among them, cDCs are subdivided into cDC1 and cDC2 subsets (108). cDC1s express toll-like receptors (TLRs) and secrete proinflammatory cytokines (IL-12p70 and IFN-α). They present endogenous antigens via MHC-I to CD8^+^ T cells, driving Th1 responses against intracellular pathogens and malignancies. cDC2s produce cytokines (IL-10 and IL-23) and present antigens to CD4^+^ helper T cells, polarizing Th2 and Th17 effector responses. This subset is expanded in patients with IBD. Soleto et al. reported that the cDC2 subset presented increased expression of homing markers (CCR6, CCR2, and β7), facilitating migration to gastrointestinal mucosa. This subset of cells may be a potential biomarker and therapeutic target (109). The DC3 subset has unique proinflammatory properties and polarizes CD8^+^ T cells into CD8^+^CD103^+^ tissue-resident memory T cells. pDCs accumulate in the colon of mice with DSS-induced colitis; suppression of pDC migration to isolated lymphoid follicles in the colon abrogates the development of colitis (110). Tolerogenic dendritic cells (tolDCs) are a special subset with immunosuppressive capabilities that can maintain immune tolerance by inducing Tregs. The latest research indicates that probiotics engage DC PRRs, modulating DC maturation and promoting tolDC generation to suppress inflammation (111). Under normal conditions, DCs modulate their barrier integrity through cytokine signaling (IL-10 and TGF-β). In IBD, DCs lose tolerogenic capacity, impairing antigen presentation and T-cell polarization. These genes exhibit upregulated TLR2/TLR4 expression in IBD, potentially driving aberrant bacterial recognition and hyperresponsiveness.

#### 3.1.3 Innate lymphoid cells

Innate lymphoid cells (ILCs), including ILC1/2/3 subsets, are crucial innate effectors that maintain barrier function in intestinal homeostasis (112). ILC1s contribute to host defense against intracellular pathogens through IFN-γ secretion, yet their dysregulated activity activates Th1-driven pathologies in CD. ILC2s mediate pathogen clearance and tissue repair via IL-5 and IL-13 production, with functional impairments strongly associated with eosinophilic gastrointestinal disorders. ILC3s display functional duality. For example, ILC3s exert protective effects by generating IL-22 to promote epithelial repair and microbiota balance (113), whereas some ILC3s secrete IL-17 and GM-CSF to activate immune networks that drive inflammatory responses (114). Microbial metabolites critically regulate ILC function. Tryptophan derivatives engage the aryl hydrocarbon receptor in ILC3s, enhancing protective cytokine secretion (115). Notably, ILC1-mediated responses are protective during acute infection but become pathological during chronic inflammation. In CD, expanded ILC1 populations mediate T-bet-dependent IFN-γ production, directly damaging the epithelium (116). In UC, decreased ILC3 counts are associate with antimicrobial peptide deficiency and barrier defects. ILC2 deficiency increases susceptibility to pathogen-induced colitis, whereas ILC3 ablation triggers spontaneous chronic inflammation in murine models (117).

#### 3.1.4 Neutrophils

As early responders of the immune system, neutrophils eliminate pathogens and participate in the control of acute inflammation, maintaining tissue integrity. In IBD, they play dual roles—both protective and pathogenic. Neutrophils secrete IL-23, which promotes ILC3 activation and tissue repair. The CD177^+^ subset of neutrophils exerts protective effects by enhancing anti-inflammatory mediators (IL-22 and TGF-β) and suppressing the levels of proinflammatory cytokines (IL-6, IL-17A, and IFN-γ), thus reducing inflammation (118). Conversely, neutrophils can also cause mucosal damage through pathogenic neutrophil extracellular traps (NETs), releasing proteases such as myeloperoxidase (MPO) and neutrophil elastase (NE), as well as reactive oxygen species (ROS) that amplify inflammation (119). Histone citrullination mediated by peptidylarginine deiminase 4 (PAD4) is a key step in NET formation (120). Inhibition of PAD4 has been shown to alleviate pathological damage in mouse models of IBD. NETs activate Toll-like receptors, trigger cytokine release (e.g., IL-1β), and induce platelet aggregation, leading to microvascular dysfunction and intestinal ischemia–reperfusion injury. Serum levels of citrullinated histone H3 and NE-DNA complexes are correlated with disease activity in IBD, suggesting that NETs may serve as noninvasive diagnostic indicators (121). NETs also release new inflammatory mediators; for example, NETs-induced secretion of IFN-γ impairs Treg function, exacerbating immune imbalance. Hypochlorous acid produced during NETs directly damages tissues, worsening histopathology in patients with CD (122). Moreover, citrullinated proteins in NETs stimulate the production of anti-neutrophil cytoplasmic antibodies, which continuously drive mucosal inflammation due to their prolonged half-life (121). Studies have shown that depleting neutrophils with neutralizing antibodies can improve colitis in animal models (118, 123). However, the complex functions of NETs in IBD remain to be elucidated. Future research should explore spatiotemporally controlled strategies to regulate NETs to balance their roles in immune defense and pathological injury.

### 3.2 Adaptive immune effectors

#### 3.2.1 T cells

The adaptive immune system plays a pivotal role in the pathogenesis of IBD, primarily through the differentiation and functional regulation of T cells. Helper T (CD4^+^) cells are classically subdivided into Th1 and Th2 subsets. Th1 cell activation, which is predominantly induced by IFN-γ, drives STAT1 phosphorylation and T-bet upregulation, thereby activating macrophages to eliminate intracellular pathogens (124). IL-12 is a critical cytokine for Th1 differentiation; binding to its receptor (IL-12R) activates STAT4 and NF-κB signaling, promoting IFN-γ, IL-12, and TNF-α production. Therapeutic targeting of IL-12 has demonstrated efficacy in ameliorating colitis. Conversely, Th2 cells produce IL-4, IL-5, and IL-13, which are essential for defense against extracellular pathogens. Naïve CD4^+^ T cells differentiate into Th17 cells under the regulation of cytokines including IL-23, TGF-β, and IL-6 (124). Th17 cells represent a distinct CD4^+^ effector lineage with significant regulatory functions in IBD. Under homeostatic conditions, they maintain immune balance and barrier defense via cytokine secretion, including microbiota modulation (125). In IBD, Th17 expansion undergoes RORγt-driven expansion and metabolic reprogramming, while also leading to the secretion of IL-17A, IL-17F, IL-21, and IL-22. This facilitates immune cell recruitment and amplifies inflammatory responses. Tissue-resident memory T (TRM) cells perpetuate chronic inflammation, and their regulation is dependent on the inhibitory receptor TIGIT (126). scRNA-seq reveals distinct T-cell subsets in CD and UC. Compared with those in UC patients, CD4^+^ and CD8^+^ TRM subsets are enriched in mucosal lesions in CD patient (127, 128), whereas CXCR5^+^ CD4^+^ T follicular helper cells predominate in UC patients (128). CD4^+^ TRM cells can adopt effector and innate-like phenotypes, directly damaging epithelial cells and exerting pathogenic effects. Their long-term persistence may drive recurrent inflammation through sustained effector activation (127).

#### 3.2.2 Regulatory T cells

Tregs maintain immune tolerance by suppressing inflammation and supporting tissue repair and homeostasis. In IBD, Tregs exhibit functional instability and may transition to proinflammatory states under cytokine pressure (108). Within the inflammatory milieu, Tregs lose their suppressive capacity (e.g., via Foxp3 downregulation) or acquire effector-like characteristics (such as a Th17-like phenotype). Loss of the nuclear receptor NCOR1 promotes effector Treg accumulation and compromises protection against intestinal inflammation (129). Intestinal Tregs display high plasticity, with phenotypes and functions modulated by inflammatory cues and metabolic stress. Notably, Clostridium clusters increase Treg accumulation, thereby suppressing gut inflammation. Impaired TGF-β signaling in IBD contributes to Treg dysfunction, which is characterized by reduced CTLA-4 expression and lineage instability (130). Emerging evidence suggests that novel inhibitory molecules regulation—including IL-35, IL-37, and miRNAs, are involved (131). Further elucidation of these regulatory mechanisms is essential for developing novel therapeutic strategies.

#### 3.2.3 B cells

B cells contribute to humoral immunity and tolerance by secreting immunoglobulin A and modulating immune responses. In IBD, B-cell dysfunction is characterized by lymphoplasmacytic infiltration and antimicrobial antibody production. Research has shown that the B-cell receptor repertoire is profoundly perturbed in IBD patients, suggesting involvement via autoantibody or abnormal immunoglobulin generation (132). Regulatory B cells suppress immunity via IL-10 secretion. In pediatric IBD, disrupted peripheral B-cell subsets and cytokine profiles—including reduced IL-10–producing B cells and increased TNF-α–producing transitional CD24hiCD38hi B cells—are partially normalized after infliximab treatment (133). IL-35 is an inhibitory cytokine of the IL-12 family, that is composed of a heterodimer of IL12a and Ebi3. IL-35-producing B cells (IL-35^+^ B cells), which are expanded in UC, promote intestinal homeostasis and disease alleviation (134). In addition, exogenous supplementation with the microbial metabolite indole-3-acetic acid further enhances IL-35^+^ B cell expansion and ameliorates colitis. For example, B-cell–depleting antibodies such as rituximab (anti-CD20) show limited efficacy in active UC, whereas obinutuzumab (a type II anti-CD20 antibody) may induce pancolitis during cancer chemotherapy (135). Alternative strategies targeting B cells or plasma cells warrant exploration.

## 4 Targeted immunomodulation: mechanistic insights and clinical translation

**Table 2** summarizes the mechanisms, clinical development stages, and potential biomarkers of targeted therapies in IBD.

**Table 2:** Summary of the mechanism, clinical stage, and potential predictive markers of immunomodulation in IBD.

Type	Drug name(s)	Target	Mechanism of action	Manufacturer	Clinical stage and ongoing evaluation	Potential predictive markers	References
Anti-TNF-α	Infliximab	TNF-α	Neutralizes TNF-α activity, reducing inflammatory responses	Johnson & Johnson	Approved for UC and CD	Anti-drug antibodies; serum drug FC; CRP	(136, 137)
	Adalimumab	TNF-α		AbbVie	Approved for CD		
IL-12/23 inhibitors	Ustekinumab	IL-12/23 p40	Binds IL-12/23 p40 subunit, inhibits Th1/Th17 differentiation and inflammatory responses	Johnson & Johnson	Approved for IBD	Serum IL-22;	(138)
IL-23 inhibitors	Guselkumab	IL-23 p19	Selectively inhibits IL-23 p19, suppresses Th17 differentiation and inflammation	Johnson & Johnson	Phase III (NCT05197049, NCT04033445)	IL-23 serum levels; genetic variants in IL23R; Th17-related gene signatures	(139)
	Risankizumab	IL-23 p19		AbbVie	Approved for CD	FC; CRP	(140)
	Mirikizumab	IL-23 p19		Eli Lilly	Phase III (NCT03518086, NCT04024092)	IL-23 pathway genes; mucosal healing score; eosinophil count	(141–143)
Integrin inhibitors	Vedolizumab	α4β7 integrin	Blocks α4β7–MAdCAM-1 interaction, inhibiting gut-specific lymphocyte migration and inflammation	Takeda	Approved for IBD	α4β7 expression on lymphocytes; MAdCAM-1 mucosal addressin; VCAM-1	(144, 145)
	Natalizumab	α4 integrin	Blocks α4 integrin, inhibits lymphocyte migration into the gut	Biogen	Approved for moderate to severe CD	JCV serostatus; α4 integrin expression	(146)
S1P receptor modulator	Ozanimod	S1P receptor	Modulates lymphocyte trafficking, reduces inflammatory cell migration	Bristol Myers Squibb	Approved for UC; Phase II/III for CD	Lymphocyte count; S1P receptor expression	(147, 148)
JAK inhibitors	Tofacitinib	pan-JAK	Inhibits JAK/STAT signaling pathway, reduces inflammation	Pfizer	Approved for UC	drug tissue concentration; pSTAT3	(149)
	Upadacitinib	JAK1		AbbVie	Approved for UC and CD	CRP	(150)
	Filgotinib	JAK1		Gilead	Approved for UC and CD	clinical scores; FC	(151, 152)
IL-6 inhibitor	PF-04236921	IL-6	Blocks IL-6 activity, reduces inflammation	Pfizer	Phase II (NCT01287897, NCT01345318)	NR	–
IL-6 trans-signaling inhibitor	Olamkicept (TJ301)	soluble IL-6R	Inhibits IL-6 trans-signaling, reduces inflammation	Ferring Pharmaceuticals	Phase II (NCT03235752)	NR	–
IL-36R inhibitor	Spesolimab	IL-36R	Blocks IL-36 receptor, reduces inflammatory responses	Boehringer Ingelheim	Phase II (NCT03482635, NCT03123120, NCT03100864)	IL-36 cytokine levels; FC	(153, 154)
TL1A inhibitors	PRA023 (Tulisokibart)	TL1A	Inhibits TL1A, reduces inflammation	Prometheus Biosciences	Phase IIa (NCT05013905, NCT04996797)	TL1A expression; genetic variants in TL1A	(155)
	PF-06480605 (RVT-3101)	TL1A		Pfizer	Phase IIa (NCT02840721)	NR	–
TYK2 inhibitor	Deucravacitinib	TYK2	Selective TYK2 inhibitor, modulates JAK-STAT signaling	Bristol Myers Squibb	Phase II (NCT03599622, NCT03934216)	NR	–
Low-dose IL-2	N/A	CD25 (IL-2R)	Expands regulatory T cells to promote immune tolerance	N/A	Phase Ib/IIa (NCT02200445)	IL-2 receptor alpha chain (CD25) expression; soluble CD25	(156)
CAR-T therapy	N/A	CD7, IL23R, etc.	Genetically modified T cells target specific antigens on immune cells	N/A	Phase I/Preclinical (NCT04691232, NCT05239702)	Target antigen density (e.g., IL23R+ cells); cytokine release profile	(157)
Fecal Microbiota Transplant (FMT)	N/A	Microbiota	Restores healthy gut microbiota composition	N/A	Multiple clinical trials (e.g., NCT01545908, NCT02390726)	Baseline microbial diversity; specific donor strain; SCFA levels	(158–160)
Microbial metabolites	N/A	N/A	Modulates barrier function and immune homeostasis via microbe-derived molecules (e.g., SCFAs, bile acids)	N/A	Preclinical	Fecal SCFAs; bile acid composition; zonulin and other gut permeability markers	(161–163)

IBD, inflammatory bowel disease; UC, ulcerative colitis; CD, Crohn’s disease; TNF-α, tumor necrosis factor-alpha; IL, interleukin; JAK, Janus kinase; STAT, signal transducer and activator of transcription; S1P, sphingosine-1-phosphate; TYK2, tyrosine kinase 2; TL1A, TNF-like ligand 1A; CAR-T, chimeric antigen receptor T-cell therapy; SCFAs, short-chain fatty acids; IEC, intestinal epithelial cell; MAdCAM-1, mucosal addressin cell adhesion molecule-1; VCAM-1, vascular cell adhesion molecule-1; JCV, JC virus; CRP, C-reactive protein; sIL-6R, soluble IL-6 receptor; Treg, regulatory T cell; Teff, effector T cell; Th1/Th17, T helper 1/T helper 17 cells; pSTAT, phosphorylated STAT; IFN-γ, interferon-gamma; FC, fecal calprotectin; N/A, not applicable. NR, no report.

### 4.1 Anti-TNFα therapy

Anti-TNF-α agents (e.g., infliximab, and adalimumab) neutralize soluble and membrane-bound tumor necrosis factor alpha (TNF-α), a master proinflammatory cytokine that drives intestinal inflammation in individuals with IBD (164). By inhibiting the binding of TNF-α to its receptor (TNFR1/2), these agents suppress downstream NF-κB signaling and reduce IL-6/IL-1β production (165). Infliximab effectively induces clinical remission in refractory UC, with some patients achieving mucosal healing (166, 167). In a Danish multicenter prospective cohort study, anti-TNF-α treatment was administered to bionaïve adult IBD patients, and the results demonstrated that over 50% of patients achieved clinical remission (168). In pediatric IBD patients, infliximab and adalimumab effectively induce and maintain steroid-free remission while reducing surgery rates (165). A meta-analysis of 17 randomized controlled trials (RCTs) involving n=8,871 confirmed the efficacy of induction and maintenance therapy, although the response was reduced in patients who switched biologics after prior anti-TNF-α exposure (169). Anti-TNF-α agents are effective at alleviating symptoms in UC patients, but their efficacy is influenced by prior drug exposure. In multicenter retrospective studies, the anti-TNF-α treatment response rate was moderate; some patients might have lost response, highlighting the importance of individual differences in the real world (170, 171).

Anti-TNF-α therapy remains a cornerstone of IBD management, exerting potent immunomodulation via T-cell depletion and myeloid silencing. Although the therapeutic effect has been confirmed, anti-TNF-α therapy faces challenges with secondary loss of response (LOR). The key factors driving LOR include the development of anti-drug antibodies (ADAs), suboptimal drug dosing or intervals, and immunogenicity. Management strategies include dose intensification, interval adjustment, immunomodulator combination therapy (e.g., azathioprine with adalimumab for CD; infliximab with immunomodulators for moderate–severe UC), or switching agents (172). As immunosuppressants, anti-TNF-α agents can increase the risk of severe/opportunistic infections, particularly when combined with other immunosuppressants, necessitating vigilant monitoring (172). Long-term use confers a slightly elevated lymphoma risk, warranting pretreatment benefit-risk assessment (170). Additional adverse effects include infusion reactions and paradoxical autoimmune reactions (e.g., drug-induced lupus). The risk in patients with prior malignancy remains uncertain (55). Therapeutic drug monitoring (TDM) optimizes outcomes by guiding treatment adjustments through measurement of drug trough levels and ADA titers, thereby enhancing treatment efficacy and improving patient prognosis.

### 4.2 IL-12/23 p40 inhibitor (ustekinumab)

Ustekinumab, a human monoclonal antibody that targets the shared p40 subunit of IL-12 and IL-23, inhibits the binding of these cytokines to their receptors (IL-12Rβ1/IL-23R) (173). This blockade suppresses Th1/Th17 cell polarization and alleviates mucosal inflammation. In patients with CD, ustekinumab effectively maintains clinical remission, including steroid-free remission, over 2 years in real-world cohorts (174), even among patients previously exposed to anti-TNF-α agents (175). Clinical trial data confirm sustained efficacy for up to 5 years (176). Ustekinumab also benefits patients with specific CD phenotypes, inducing stenosis regression or improvement in 62.5% of patients with fistulizing disease and subsiding perianal disease in 38.5% of patients with stenosing disease (177). In UC, ustekinumab has demonstrated efficacy for short- and long-term clinical improvement in adults (178, 179), including anti-TNF-α-refractory pediatric patients (180). Its long-term safety profile is favorable, with rates of serious infections and malignancies comparable to those of placebo groups across 5-year (CD) and 4-year (UC) trials (176).

### 4.3 IL-23 p19 inhibitors (guselkumab, risankizumab, mirikizumab)

Compared with p40 inhibitors (e.g., ustekinumab), IL-23 p19 inhibitors selectively target the IL-23 p19 subunit, preserving IL-12–mediated immune surveillance and reducing opportunistic infection risk (181). These agents demonstrate a lower adverse event risk (relative risk: 0.79, 95% CI: 0.61–1.02) that do anti–IL-12/23 agents (182). In a head-to-head trial of moderate-to-severe CD, risankizumab achieved superior clinical remission at week 24 (58.6% vs. 39.5%) and endoscopic outcomes at week 48 (31.8% vs. 16.2%) compared with ustekinumab (183). **Table 3** lists the clinical research progress and long-term efficacy of IL-23 p19 inhibitors.

**Table 3:** Clinical research progress and long-term efficacy of IL-23 p19 inhibitors.

Drug	Indications	Core clinical data	Study source
Mirikizumab	UC, CD	UC: 40-week follow-up: 49.9% clinical remission vs. placebo (25.1%) (184); CD: 52-week follow-up: 45.4% CDAI clinical remission vs. placebo (19.6%), 38% endoscopic remission vs. placebo (9.0%) (185).	LUCENT-1 and LUCENT-2 (NCT03518086 and NCT03524092)
			VIVID-1 (NCT02589665)
Guselkumab	UC, CD (Phase III)	CD: 44-week clinical remission rate vs. placebo (Δ=48.9%, *P* < 0.01), endoscopic remission vs. placebo (Δ=44.6%, *P* < 0.01) (186); UC: 44-week clinical remission rate vs. placebo (Δ=30%, *P* < 0.01) (187).	GRAVITI (NCT05197049); QUASAR (NCT04033445).
Risankizumab	CD (Approved)	CD: 48-week endoscopic response rate 31.8% (vs ustekinumab 16.2%) (183); CD: 52-week: CDAI clinical remission vs. placebo (Δ=15%, *P* < 0.01), endoscopic remission vs. placebo (Δ=28%, *P* < 0.01).	NCT04524611; NCT03105102.

UC, ulcerative colitis; CD, Crohn’s disease; CDAI, Crohn′s disease activity index.

IL-23 p19 inhibitors retain efficacy in patients with prior anti-TNF-α therapy failure, highlighting their therapeutic utility across biologic classes (188). All of these compounds exhibited favorable safety profiles, with serious adverse events and infection rates comparable to those of the placebo groups. Single-cell transcriptomics implicates inflammation-associated fibroblasts in anti-TNF-α resistance, while *IL1B* drives vedolizumab unresponsiveness (189). In anti-TNF-α–refractory severe CD, dual targeting of IL-23p19 and IL-1β may confer clinical benefit (190). Currently, no IL-23p19 inhibitors are approved for use in pediatric IBD patients, highlighting the urgent need for prospective safety studies. Additionally, Ota et al. developed orally administered anti–IL-23R VHH antibodies that show promise—offering intestinal stability and non-systemic delivery to overcome injection limitations in preclinical studies (191). Prospective safety studies are urgently needed.

### 4.4 Anti-integrin (vedolizumab, natalizumab)

Vedolizumab, the first anti-integrin approved for IBD, selectively inhibits gut lymphocyte migration and associated inflammation by blocking the α4β7 integrin–MAdCAM-1 interaction (192). The GEMINI long-term safety study demonstrated sustained efficacy and safety in moderate-to-severe UC and CD patients, with up to 5 years of follow-up showing infection and malignancy rates comparable to those of placebo (193). Long-term clinical remission rates of 40%–50% were achieved during maintenance therapy for both CD and UC patients, including patients refractory to conventional therapy or anti-TNF-α agents (192). In routine practice, vedolizumab has higher remission rates and a lower incidence of serious adverse events than TNF-α antagonists in TNF-α-naïve UC patients (194). Mechanistically, vedolizumab blocks the migration of activated T cells—including pro-inflammatory Th1 and Th17 subsets—from the vascular endothelium to the intestinal mucosa, thereby reducing T-cell-mediated inflammation (195). It reduces naïve B and T cells in the gut and significantly decreases the number of circulating gut-homing (β7^+^) plasmablasts in UC patients (196). Zeissig et al. found that vedolizumab does not alter the intestinal T-cell receptor repertoire or the relative abundance of various lamina propria T-cell subsets (e.g., CD4^+^, CD8^+^, central, and effector memory T cells) (197). Further research is needed to clarify its mechanisms.

Natalizumab (anti-α4 integrin) is approved for moderate-to-severe CD, but its use is limited because of the potential risk of progressive multifocal leukoencephalopathy (PML), particularly in patients positive for JC virus antibodies (146). To mitigate this risk, strict patient selection is needed, which is limited to treatment-refractory cases with mandatory requirements (146). Additionally, active monitoring through regular MRI scans and neurological assessments is recommended, with short-term use advised for seropositive patients (198). Owing to the risk of systemic immunosuppression, natalizumab has been largely replaced in IBD by gut-selective vedolizumab and is now primarily used to treat multiple sclerosis (199). However, natalizumab may still be considered for the rapid induction of remission, while vedolizumab is preferred for long-term maintenance.

### 4.5 Ozanimod (S1P receptor modulator)

Ozanimod is the first oral S1P receptor modulator approved for UC. In phase III UC trials, ozanimod demonstrated superiority over placebo, with clinical remission rates of approximately 18.4% (induction) and 37% (maintenance) (200). Common adverse events include transient lymphopenia, elevated liver enzymes, and headache; the risk of severe infection is lower than that associated with systemic immunosuppressants (201). For CD, phase II/III data suggest the potential for remission, although approval remains pending (200).

Both the integrin and S1P modulator classes inhibit lymphocyte migration. Integrin inhibitors (vedolizumab) selectively block gut homing (α4β7-MAdCAM-1), whereas S1P modulators (ozanimod) systemically inhibit lymphocyte egress (202). S1P modulators may cause transient heart rate reduction but carry no risk of PML; integrin inhibitors offer greater gut selectivity with fewer systemic effects (203). Oral S1P modulators provide novel UC treatment options with a broader mechanism of action, necessitating cardiovascular monitoring at initiation (203).

### 4.6 JAK inhibitors

Biological agents require intravenous or subcutaneous injection and are prone to triggering ADA production because of their significant immunogenicity. In contrast, small-molecule drugs (molecular weight <1 kDa, typically <500 Da) diffuse across cell membranes into the cytoplasm, generally lack immunogenicity, and offer advantages including oral bioavailability, convenient dosing, structural stability, lower manufacturing costs, shorter half-lives, and rapid elimination.

Janus kinase (JAK) inhibitors target intracellular tyrosine kinases (JAK1–3 and TYK2). Tofacitinib, a pan-JAK inhibitor, lacks selectivity and is associated with significant off-target toxicities, including malignancy and venous thromboembolism (204). Nevertheless, it remains an effective treatment for moderate-to-severe UC, particularly in patients failing or intolerant to biologics, with proven efficacy in inducing and maintaining clinical remission in phase III trials (205). Upadacitinib and filgotinib are selective JAK-1 inhibitors that have been approved for the treatment of UC. In the phase III U-ACHIEVE and U-ACCOMPLISH trials, upadacitinib induced clinical remission in 26% of patients at week 8 compared with 5% with placebo, and maintained remission in 42% of patients at week 52 (206). In CD, phase II/III studies have shown improved remission rates, although approval is pending (207). Most JAK inhibitors (except upadacitinib) have shown limited efficacy in CD, potentially owing to heterogeneity in inflammatory pathways. All JAK inhibitors are contraindicated during pregnancy and lactation because of the potential embryotoxicity observed in animal studies (208). To mitigate systemic toxicity, gut-selective JAK inhibitors such as TD-1473 represent a promising novel strategy (209). Deucravacitinib, a selective TYK2 inhibitor was evaluated in three randomized and double-blind phase II trials (LATTICE-CD, LATTICE-UC, and IM011-127) for moderate-to-severe active CD and UC (210). Although it was well tolerated across multiple doses (3 mg, 6 mg, and 12 mg), it did not yield significant clinical benefits over the placebo in either CD or UC patients.

### 4.7 Anti-TL1A inhibitors

Anti-TL1A therapy targets the Th1/Th17 pathways, demonstrating potential for mucosal healing in IBD (211). PRA023 (tulisokibart), a humanized anti-TL1A IgG1-κ monoclonal antibody, has shown promising efficacy in IBD. In a phase IIa trial involving CD patients (NCT05013905), tulisokibart induced endoscopic remission in 26% of participants, with 49.1% achieving clinical remission at week 12 (212). Similarly, in a phase 2 trial for moderate-to-severe UC (NCT04996797), 26% of tulisokibart-treated patients achieved clinical remission vs. 1% with placebo; endoscopic remission was 37% vs. 6.0% at week 12 (155). Another agent, PF-06480605 (RVT-3101), is a fully humanized TL1A-directed IgG1 monoclonal antibody with high affinity and specificity. In the phase 2a TUSCANY trial (NCT02840721), PF-06480605 treatment resulted in 43% clinical remission and 64% endoscopic remission by week 56 (213). Fecal microbiome analysis further revealed a significant reduction in IBD-associated pathogenic bacteria. These compelling outcomes underscore the therapeutic potential of anti-TL1A agents and support the expansion of clinical evaluations in IBD.

### 4.8 IL-36 inhibitors

The IL-36 signaling pathway has recently emerged as a critical mediator in maintaining intestinal homeostasis and modulating inflammatory responses. Spesolimab, a newly developed humanized monoclonal antibody, specifically inhibits the IL-36 pathway. In a series of phase II/IIa clinical trials (NCT03482635, NCT03123120, and NCT03100864), Ferrante et al. evaluated the safety and therapeutic potential of spesolimab in patients with UC (214). Although the drug was found to be generally safe and well-tolerated, its clinical effectiveness was limited, with no significant difference compared with that of the placebo, and only 14.3% of patients showed endoscopic improvement; however, Hecker et al. recently reported that spesolimab is effective for CD patients with IL36RN mutations, suggesting that inhibiting IL-36 may provide a personalized treatment option for this subgroup (215).

### 4.9 IL-6 cross-signaling inhibitors

In IBD, IL-6 levels are significantly elevated and strongly correlated with disease activity, relapse, and inflammation severity. Although the anti-IL-6 antibody PF-04236921 improved the clinical response and remission in moderate-to-severe CD patients, it caused immune-related adverse events such as abscesses and perforations, possibly due to the protective role of IL-6 in gut homeostasis (216). Blocking IL-6R effectively suppresses intestinal inflammation. Olamkicept (TJ301, FE 999301), a first-in-class soluble IL-6 receptor (sIL-6R)/IL-6 complex inhibitor, specifically blocks IL-6 trans-signaling. In a randomized phase 2 trial, 58.6% of active UC patients responded clinically after 12 weeks of biweekly 600 mg infusions, whereas 34.5% responded with placebo, indicating strong therapeutic potential (217).

### 4.10 Low-dose IL-2

Low-dose IL-2 (LD-IL-2) therapy aims to restore Treg-mediated tolerance while avoiding global immunosuppression. To assess the safety and tolerability of LD IL-2, a phase 1b/2a clinical trial was implemented in patients with moderate-to-severe UC (218). LD IL-2 was well-tolerated, with no serious adverse effects (AEs) and no deaths. The Mayo endoscopic score at 8 weeks revealed that 69.2% of the patients achieved a clinical response and 30.8% achieved clinical remission.

### 4.11 Treg adoptive therapy

Treg adoptive therapy aims to restore immune homeostasis in the inflamed gut by isolating, expanding, and reinfusing autologous Tregs to specifically suppress pathogenic effector T cells, particularly Th17 cells, thereby rebalancing the dysregulated Th17/Treg axis. Voskens et al. published data (NCT04691232) on one patient with refractory UC and associated primary sclerosing cholangitis (PSC) receiving adoptive Treg therapy (a single infusion of 1.0×10^6^ Tregs/kg body weight) (219). After adoptive Treg transfer, the Mayo score decreased from 8 points on the day of transfer to 4 points at the 12th week of follow-up. A phase 1 clinical trial (NCT05239702) is currently underway to assess the safety and effectiveness of CD7 CAR-T cell infusion in patients with autoimmune diseases, including CD and UC. Cui et al. designed Tregs expressing CAR-IL-23R, generating IL-23R-CAR-Tregs for treating CD (220). The experimental results showed that the infusion of IL-23R-CAR-Treg cells could protect mice from experimental colitis. Although early-phase clinical trials have demonstrated their safety and preliminary efficacy, key challenges remain: inefficient homing of infused Tregs to intestinal sites of inflammation, limited understanding of migratory mechanisms, and difficulties in expanding antigen-specific Tregs without causing nonspecific immunosuppression.

### 4.12 Gut microbiota and microbial metabolites

The maintenance of host physiological functions relies heavily on the essential contribution of the gut microbiota. Notably, IBD patients exhibit microbial dysbiosis, characterized by reduced microbial diversity, a decrease in anti-inflammatory anaerobic bacteria, and an increase in proinflammatory bacterial species (221). Probiotic supplementation and fecal microbiota transplantation (FMT) have shown potential in ameliorating colitis by modulating the microbial composition (222). FMT involves transplanting the intestinal microbiota from healthy donors to patients to restore microbial balance. Several RCTs have demonstrated that FMT can induce remission in mild to moderate UC (NCT01545908, NCT02390726) and CD patients (NCT02097797). However, endoscopic remission rates and long-term maintenance of remission remain challenging. Nevertheless, studies indicate that FMT is effective and safe in pediatric UC patients with cytomegalovirus-induced colitis (223) and in IBD patients with recurrent Clostridium difficile infection (224). Although FMT can induce remission in patients with IBD, its efficacy varies across individuals, potentially due to differences in the stability and metabolic activity of the donor microbiota (158). Preconditioning donor mice with the plant L. plantarum GR-4 can increase the stability of the donor microbiota and thereby improve the efficacy of FMT (158). Compared with conventional FMT, whole intestinal microbiota transplantation allows more accurate replication of the donor microbiota and reduces host inflammatory responses (225). However, the effectiveness of oral probiotics remains limited by poor survival and colonization rates in the gut. Currently, the American Gastroenterological Association recommends that FMT be used only within clinical trials for IBD pending further validation of its safety and efficacy (226). The long-term effects of microbiota transplantation, donor screening criteria, and immune interaction mechanisms remain unclear. The role of viral components in FMT and their influence on inflammatory regulation also warrant further investigation (227). Future research should focus on identifying microbial markers—such as specific bacterial strains or metabolic pathways—associated with FMT treatment response (228). The development of standardized FMT preparations (e.g., lyophilized fecal microbiota) or targeted probiotic combinations may increase treatment precision (229). While FMT and probiotics represent promising therapeutic options for IBD, their application requires optimization through larger-scale clinical studies and mechanistic exploration.

Microbial metabolites, including short-chain fatty acids (SCFAs), bile acids, and indole derivatives, help regulate intestinal barrier integrity and immune homeostasis (230, 231). In IBD patients, these metabolites are often dysregulated; for example, sulfonolipid biosynthetic enzymes are downregulated (232), whereas bacterial siderophores and genotoxins directly contribute to IBD pathogenesis (233). Succinate, a microbiota-derived metabolite, promotes UC-related inflammation by activating IL-9-producing helper T cells (234). Conversely, SCFAs such as butyrate alleviate inflammation by enhancing Treg cell function (161). Clostridium butyricum exerts anti-inflammatory effects by increasing the colonic levels of SCFAs and retinoic acid, thereby improving symptoms in murine colitis models (162). This bacterium also enhances intestinal barrier function in inflammatory depression models, reduces aberrant metabolite accumulation, and exhibits antidepressant-like effects in mice (163). Additionally, tungsten ion-loaded mesoporous polydopamine-coated Lactobacillus acidophilus has been shown to modulate lipid metabolism via the gut–liver axis, scavenge reactive oxygen and nitrogen species, and ameliorate colitis while restoring gut–liver homeostasis (235). Compared with broad-spectrum interventions, targeted supplementation with specific metabolites—such as personalized microbiota-metabolite cosupplement strategies—has greater therapeutic potential (236).

### 4.13 Nanomedicine

Nanotherapeutics leverage nanoparticles (micelles, liposomes, or polymeric nanoparticles) to achieve precise, site-specific drug delivery, overcoming the limitations of conventional IBD therapies (237). This advantage stems from the unique physicochemical properties of nanoparticle-based drug delivery systems, which enhance permeability and retention effects to facilitate targeted drug deposition at inflammatory sites (238). Inorganic nanoparticles or nanomaterial-based natural products can mimic biological enzyme activity to effectively neutralize ROS/RNS, thereby mitigating inflammation (239, 240). Functional surface modifications of polymeric nanoparticles, such as pH/redox dual-responsive butyrate-rich polymer nanoparticles, enhance cellular targeting and biosafety, demonstrating pronounced mucosal targeting efficacy in preclinical studies (241). Wang et al. reported that oral precision nanomedicine demonstrated effects in reducing inflammation and improving intestinal function in a murine model of colitis (242). Despite their potential in IBD, nanomedicine faces practical challenges in translating from experimental models to the clinic, including biocompatibility, long-term safety, and scalable manufacturing of nanocarriers, as well as incomplete knowledge of their interactions with the gut mucosal interface. Future multifunctional nanoplatforms, designed to exploit the pathological features of the gut microenvironment and integrate drug delivery, mucosal repair, and theranostics will be key to addressing the complex pathogenesis of IBD (241).

## 5 Toward precision medicine: integrating genetics, immunology, and therapeutics

### 5.1 Limitations of existing biomarkers

Biomarker research in IBD has advanced significantly toward non or minimally invasive solutions for diagnostic, prognostic, and treatment options. However, key challenges remain. C-reactive protein (CRP) and fecal calprotectin (FC) remain frontline tools for IBD diagnosis and monitoring, distinguishing IBD from IBS but lacking specificity for differentiating CD from UC (243). Spatiotemporal variability in inflammation and treatment response complicates biomarker validation. Dynamic disease states necessitate integrative biomarker signatures that reflect global pathology; however, current markers fall short in capturing this complexity (244). Noninvasive tests also lack the anatomical precision of endoscopy and are less accurate or cost-effective than desired. Anti-TNF-α therapies fail in 30–50% of patients, highlighting the lack of reliable predictors of response (244). The variability of real-world data limits reproducibility (245). In pediatric populations, diagnostic delays increase the risk of complications like growth impairment; yet validated early biomarkers are lacking (246). Current biomarkers also poorly predict complications such as strictures. Emerging signatures such as volatile organic chemicals (247), lncRNAs (248, 249), and lipidomics (250) face challenges in standardization, scalability, and cost-effectiveness. Although omics approaches have identified potential predictors, the complexity of omics data analysis further limits their use in routine clinical practice (251). Future efforts must focus on standardized multicenter validation, pediatric-specific development, and simplified analytical workflows to advance precision medicine in IBD (252).

### 5.2 Predictive potential of susceptibility genes and expression profiling in IBD

Susceptibility gene and gene expression profile analyses offer promising avenues for predicting IBD risk, phenotype, and prognosis. Susceptibility genes, particularly those identified through GWAS, enable IBD risk stratification via polygenic risk scores (PRSs). PRS models leveraging high-impact variants show strong predictive accuracy in large cohorts, facilitating early screening (253). The TWAS further improves risk prediction by integrating genetic and expression data, identifying 186 novel candidate genes—such as ETS2 and PTPN2—with roles in macrophage regulation and intestinal immunity (6). Notably, the IBD-associated PTPN2 variant rs1893217 increases susceptibility to SARS-CoV-2 infection, underscoring its relevance in predicting the risk of for multiple diseases (254). Large-scale population studies identify blood protein signatures associated with IBD susceptibility (255).

The phenotypes of IBD, including disease subtypes, activity and anatomical extent are highly heterogeneous. Expression profiling effectively classified these diverse phenotypes. Differential gene expression in intestinal biopsies distinguishes disease activity: 84 dysregulated genes characterize active pancolitis, whereas quiescent disease shows fewer differences (256). Genetic risk scores combined with clinical variables can be used to differentiate UC and CD subtypes, supporting tailored therapy selection (257). scRNA-seq and spatial transcriptomics reveal cell-specific signatures; for example, cuproptosis-related genes in epithelial/immune cells are correlated with mucosal inflammation and progression in UC (258, 259). Tissue-specific eQTL analyses identified 190 inflammation-dependent regulatory loci that improve molecular subtyping (15). Phenotype-associated genes—such as the CD-protective gene IL1RL1 and the UC risk genes GPX1, GPBAR1, and PNMT—represent promising predictive targets according to a Mendelian randomization study (260). Gene expression profiling predicts treatment response and disease progression. Pretreatment transcriptomic profiles can predict anti-TNF-α efficacy; longitudinal analyses in pediatric IBD patients can identify early molecular responders (261). For CD progression risk, rectal biopsy-derived transcriptomic risk scores demonstrate high positive predictive value (262).

### 5.3 Immune cell-based biomarkers: diagnostic, phenotyping, and predictive utility

Immune cell profiling in both peripheral blood and intestinal tissue holds significant potential for advancing personalized management of IBD. For example, immunophenotypic analysis of 39 circulating immune cell subsets revealed that an increase in CD45RA⁻ CD4^+^ T cells coupled with a reduction in total T cells can distinguish between CD and UC (263). Furthermore, high-dimensional flow cytometry examining 59 immune cell subsets has facilitated the development of gut-specific immune signatures that quantify immune dysregulation and evaluate the severity of small intestinal inflammation (264). Specific cellular alterations—such as abnormalities in Paneth cell phenotypes in the ileum—have been identified as cellular markers reflecting genetic and environmental influences on CD pathogenesis (265). In colorectal cancer associated with IBD, the presence of abundant IL-22-producing tumor-infiltrating immune cells has been correlated with improved overall survival (266). scRNA-seq has further elucidated disease-specific mechanisms, including IL-1β-driven inflammation triggered by “high-damage” bacterial strains in UC, suggesting novel tools for molecular subtyping (267). Additionally, neutrophil-derived markers such as the CD64 index provide more direct measures of acute inflammation than conventional biomarkers such as CRP or FC (268).

Immune cell ratios, including the NLR and PLR, also have clinical utility in diagnosing IBD and assessing disease activity. For example, decreases in the NLR and PLR were observed at baseline in UC patients who achieved clinical and endoscopic remission following 54 weeks of anti-TNF-α therapy (269). Moreover, elevated frequencies of CD4^+^α4β7^+^ T cells at baseline are associated with the response to vedolizumab in UC patients, whereas CD4^+^α4β1^+^ T-cell subsets may predict vedolizumab efficacy in CD patients by week 14 (8, 270). For therapies targeting IL-23p19, features related to Th17 biology—such as IL-17A secretion and STAT pathway activation—represent promising candidate biomarkers. The Treg/Th17 balance is also mechanistically implicated in the treatment response; however, prospective clinical validation remains necessary.

### 5.4 Personalized drug selection in IBD: genetic and immune signatures

Genetic markers are highly important for significance in predicting the response of IBD patients to specific drugs. HLA-DQA1*05 allele carriage increases the risk of immunogenicity (ADAs) and secondary loss of response to infliximab or adalimumab (271). Pediatric cohorts highlight that certain SNPs are associated with the early response and immunogenicity of anti-TNF-α therapies (272). IL23R variants may affect the sensitivity of patients to ustekinumab treatment.

Immune characteristics such as Th17 cell activity and the abundance of specific macrophage subsets are also significant predictors of treatment response in IBD (189). The spatial distribution of macrophages/T cells in inflamed mucosa independently predicts the response to checkpoint inhibitors. Elevated baseline immune activity, particularly increased activation of the Th17 pathway or increased macrophage abundance, is associated with a reduced anti-inflammatory response. Checkpoint molecule LAG-3 expression and genetic variants may refine patient stratification for novel biologics (273). Assessing these immune features helps identify patients who may respond to specific treatment regimens.

TDM is a critical tool in the management of IBD with biological agents. It is widely used in clinical practice to assess treatment responses to anti-TNF-α agents such as infliximab and adalimumab. Low trough levels (e.g., infliximab < 3 μg/mL) predict secondary non-response, and dose escalation improves drug durability (271). The combination of TDM and the detection of ADAs helps differentiate primary nonresponse from immunogenic failure, which can be managed by a drug switch (274).

**Figure 2:**
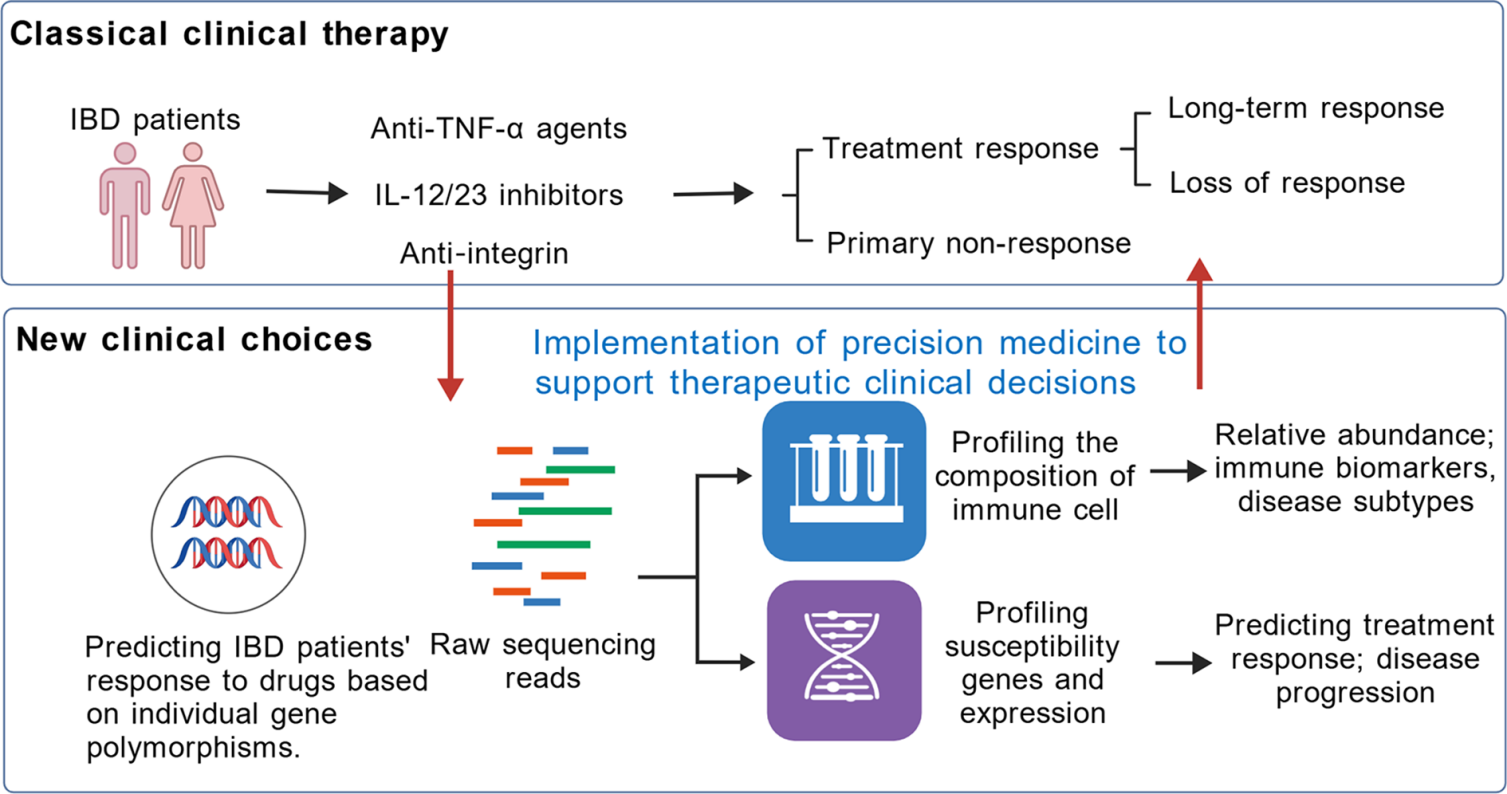
Precision medicine offers a promising strategy for IBD management through multi-omics and emerging technologies to promote biomarker development, enabling the prediction of drug responses and personalizing treatment. This construct was created with BioGDP.com (23). IBD: inflammatory bowel disease; TNF-α: tumor necrosis factor-alpha; IL: interleukin.

With an in-depth understanding of the molecular mechanisms of IBD, multiomics integration models have emerged as key directions for future research. Integrating genomics, transcriptomics, proteomics, and microbiome data can enable the development of robust predictive algorithms (275). They can provide more comprehensive information on disease characteristics, thereby enhancing the accuracy of predicting treatment response. Spatial multiomics approaches, such as digital spatial profiling, allow for the dissection of cell-type-specific resistance mechanisms within complex tissue microenvironments (276). Artificial intelligence (AI) also holds significant promise for the advancement of personalized IBD treatment (275). Machine learning models can integrate diverse patient data—including clinical features, demographic information, genomic profiles, and multiomics datasets—to predict treatment response and disease progression (277). AI can also be used to optimize treatment plans, reduce unnecessary drug trials, and improve treatment efficiency (278). These findings provide a theoretical basis for personalized treatment (**Figure 2**).

## 6 Discussion

The pathogenesis of IBD is complex and multifactorial, involving intricate interactions among various immune cells and the dysregulation of key signaling pathways. The immunoregulatory network plays a central role in IBD pathophysiology, particularly through the activation and functional modulation of TRM cells in the intestinal mucosa, which significantly influences the chronic inflammatory characteristics of the disease (279). In addition, genetic factors are implicated in IBD susceptibility. GWASs have identified numerous genetic variants associated with IBD, providing crucial insights into dysregulated immune pathways and impaired intestinal barrier function. Contemporary therapeutic strategies, such as monoclonal antibodies targeting IL-12/23 and JAK inhibitors, significantly improve clinical symptoms and patients’ quality of life by modulating these dysregulated mechanisms.

Despite substantial progress in IBD therapeutics, several challenges remain. First, the considerable heterogeneity in disease manifestations and treatment responses among patients complicates the implementation of personalized therapeutic approaches (280). Second, the functional plasticity of immune cells during disease progression introduces additional layers of therapeutic complexity (280). Moreover, the current lack of validated biomarkers capable of reliably predicting disease course and therapeutic response hinders the implementation of precision medicine. Long-term immunosuppressive therapy is also associated with risks, including diminished efficacy owing to drug resistance and adverse events such as increased susceptibility to infections and potential oncogenic effects. Consequently, although recent therapeutic advances have improved patient outcomes, achieving sustained remission or a definitive cure remains a significant challenge.

The interplay between the neuroimmune, metabolic-immune, and epigenetic-immune axes plays a pivotal role in IBD pathogenesis. Research highlights the critical importance of gut microbiota–host interactions in maintaining intestinal immune homeostasis (281). Furthermore, epigenetic modifications—including DNA methylation and histone alterations—have crucial regulatory influences on gene expression and immune responses in IBD (282, 283). Future research should prioritize the elucidation of these complex regulatory networks to inform the development of novel therapeutic strategies. Advances in scRNA-seq and spatially resolved transcriptomics have enabled researchers to precisely map the dynamic spatiotemporal interactions of immune cells within the intestinal microenvironment (284). These methodologies deepen the understanding of IBD immunopathology and enable identification of specific immune cell subsets and their interactions for more targeted therapies (285).

Due to their mechanisms and dynamic responses to the local microenvironment, TRM cells have shown promising prospects as therapeutic targets in IBD (286, 287). However, current treatment strategies targeting TRMs remain largely theoretical, with limited direct evidence of their clinical efficacy. Future research should elucidate the regulatory network of TRMs and refining targeted approaches to improve IBD treatment. While conventional immunosuppressive therapies can alleviate IBD symptoms, their long-term efficacy is often limited by drug resistance and significant adverse effects. As a result, there is increasing focus on developing “beyond-suppression” strategies, such as enhanced Treg therapies (52) and tolerogenic DC vaccines (288), which aim to restore immune tolerance and promote tissue repair. Additionally, microbiota-based interventions—including next-generation probiotics (289) and FMT (222)—represent promising emerging approaches for restoring gut homeostasis and improving therapeutic outcomes, warranting further clinical investigation. The emergence of precision medicine has accelerated the implementation of personalized therapeutic strategies in IBD management. Integrating genomics, proteomics, and other multiomics profiling technologies enables a more accurate assessment of individual immune status and disease risk, thereby supporting the development of tailored treatment regimens (290). Moreover, multidisciplinary combinatorial approaches—such as integrating immune cell therapy with chemotherapy or traditional Chinese herbal medicine—hold promise for enhancing treatment efficacy while minimizing adverse events (291, 292).

## 7 Conclusion

Advances in fine-mapping, functional genomics, and single-cell multiomics have identified pathogenic mutations within risk loci and revealed novel genes with significant implications for immune cell function in IBD. The current therapeutic landscape for IBD encompasses cytokine blockade (e.g., IL-12/23, IL-23), intracellular signaling inhibition (JAK-STAT), and emerging approaches. While IL-23-targeted biologics offer enhanced cellular selectivity and JAK1 inhibitors exhibit strong efficacy, their safety profiles demand personalized risk–benefit assessments. Promising emerging strategies—such as FMT, microbial metabolite-based therapies, and nanodelivery systems—hold transformative potential but require rigorous clinical validation. Future progress will depend on the integration of genetic predictors, TDM, and mechanistic synergy to navigate an increasingly complex treatment paradigm. Despite the shift from broad immunosuppression toward precision biologics and cellular interventions, the inherent heterogeneity of IBD remains a major challenge. Prioritizing fundamental research into immune regulatory mechanisms, developing precision therapeutics, and identifying novel biomarkers and diagnostic tools will be essential to achieve sustained remission and ultimately cure IBD.
